# *col1a2^+^* fibroblasts/muscle progenitors finetune xanthophore countershading by differentially expressing *csf1a/1b* in embryonic zebrafish

**DOI:** 10.1126/sciadv.adj9637

**Published:** 2024-04-05

**Authors:** Jiahao Chen, Honggao Wang, Shuting Wu, Ao Zhang, Zhongkai Qiu, Peng Huang, Jianan Y. Qu, Jin Xu

**Affiliations:** ^1^ Department of Neurology, the Second Affiliated Hospital, School of Medicine, South China University of Technology, Guangzhou 510006, China.; ^2^ Innovation Centre of Ministry of Education for Development and Diseases, School of Medicine, South China University of Technology, Guangzhou 510006, China.; ^3^ Department of Neurobiology, Harvard Medical School, 220 Longwood Avenue, Boston, MA 02115, USA.; ^4^ Division of Life Science, State Key Laboratory of Molecular Neuroscience, Hong Kong University of Science and Technology, Clear Water Bay, Kowloon, Hong Kong, PRC.; ^5^ Department of Biochemistry and Molecular Biology, Alberta Children’s Hospital Research Institute, Cumming School of Medicine, University of Calgary, Calgary, AB T2N 4N1, Canada.; ^6^ Department of Electronic and Computer Engineering, The Hong Kong University of Science and Technology, Kowloon, China.

## Abstract

Animals evolve diverse pigment patterns to adapt to the natural environment. Countershading, characterized by a dark-colored dorsum and a light-colored ventrum, is one of the most prevalent pigment patterns observed in vertebrates. In this study, we reveal a mechanism regulating xanthophore countershading in zebrafish embryos. We found that *csf1a* and *csf1b* mutants altered xanthophore countershading differently: *csf1a* mutants lack ventral xanthophores, while *csf1b* mutants have reduced dorsal xanthophores. Further study revealed that *csf1a* is expressed throughout the trunk, whereas *csf1b* is expressed dorsally. Ectopic expression of *csf1a* or *csf1b* in neurons attracted xanthophores into the spinal cord. Blocking *csf1* signaling by *csf1ra* mutants disrupts spinal cord distribution and normal xanthophores countershading. Single-cell RNA sequencing identified two *col1a2^+^* populations: *csf1a^high^csf1b^high^* muscle progenitors and *csf1a^high^csf1b^low^* fibroblast progenitors. Ablation of *col1a2^+^* fibroblast and muscle progenitors abolished xanthophore patterns. Our study suggests that fibroblast and muscle progenitors differentially express *csf1a* and *csf1b* to modulate xanthophore patterning, providing insights into the mechanism of countershading.

## Teaser

Countershading of xanthophores in zebrafish embryos is modulated by *csf1a* and *csf1b* expressing fibroblast and muscle progenitors.

## INTRODUCTION

Animal traits and pigment patterns have diverse biological functions, including ultraviolet (UV) protection, camouflage, mate choice, species recognition, and sexual selection. Vertebrates have evolved an extraordinary variety of pigment patterns, achieved by selectively expressing pigment genes and modifying pigment cell distribution and size. Pigment patterns are tailored to specific ecological niches and life histories and provide valuable insights into the evolutionary processes that have led to the diversity of life on earth (*1*–*10*).

Pigment cells in vertebrates are derived from neural crest cells, a multipotent cell type that emerges at the border of the neural plate (*11*, *12*). While homeothermic vertebrates such as mammals and birds have only one type of pigment cells, namely, melanocytes, which generate various melanin pigments and are responsible for forming the pigment pattern (*13*, *14*), ectothermic vertebrates including fish, reptiles, and amphibians have developed several types of pigment cells known as chromatophores. These include black melanophores, yellow xanthophores, iridescent iridophores, white leucophores, red erythrophores, and blue cyanophores (*15*–*21*). Despite the variety of pigment patterns in both homeothermic and ectothermic vertebrates, they can be classified into three major types: dorsal-ventral patterning, stripes, and spots (*22*). The dorsal-ventral patterning is usually expressed as a dark-colored back and a light-colored abdomen which is called the countershading (*23*). Countershading is one of the most widespread adaptations among vertebrates. In mammals, Agouti signaling is a dominant regulator of countershading, controlling the melanin types synthesized by melanocytes. Agouti signaling protein (ASIP) expression is strictly restricted to the ventrum, suppresses melanocyte maturation and pigmentation, and consequently leads to a light-colored ventrum in mouse embryos (*2*). As the receptor of ASIP, melanocortin 1 receptor (MC1R) is also essential for pigmentation of melanocytes (*24*). *Tbx15* expression is restricted to the dorsal mesenchyme and is complementary to Agouti expression. Loss of *Tbx15* expression leads to the dorsal expansion of Agouti expression and the transformation of dorsal hair from black to yellow (*25*).

Nowadays, zebrafish has emerged as a valuable model organism for investigating the genetic and cellular mechanisms that underlie the formation of pigment patterns. Adult zebrafish is well recognized for its horizontal-stripe patterning, which is formed by the spatial organization of three kinds of chromatophores: melanophores, iridophores, and xanthophores (*17*, *26*). On the other side, countershading is also evident in adult zebrafish as evidenced by the avoidance of melanophores and xanthophores on their ventral surface. Similar to mammals, countershading relies on the Asip/Mc1r signaling in adult zebrafish (*27*–*29*). This pathway not only regulates melanocyte pigmentation but also influences the numbers of different types of chromatophores. By shedding light on the mechanisms of countershading, these studies have paved the way for further investigation into this phenomenon.

We found that the sparsely distributed xanthophores in embryonic zebrafish establish a countershading pattern, with the dorsum of the embryo exhibiting a more yellow hue compared to the ventrum. Despite this observation, the mechanism underpinning xanthophore countershading in zebrafish remains elusive. Proper spatial localization and migration of xanthophores are crucial for pigment pattern formation in adult fish (*21*, *30*, *31*). Colony-stimulating factor 1 receptor a (Csf1ra) which encodes a tyrosine kinase receptor was known to regulate the survival and migration of xanthophores in zebrafish. Csf1ra deficiency results in the failure of pigment pattern formation in both embryonic and adult zebrafish (*32*). Zebrafish has three ligands of Csf1ra, including Csf1a, Csf1b and Il34, with previous studies indicating that *csf1a* and *csf1b* are involved in xanthophore development (*30*, *33*). However, their specific mechanisms in countershading have not yet been investigated.

Fibroblasts have conventionally been acknowledged for their role in synthesizing, remodeling, and depositing extracellular matrix, thereby providing structural support to tissues and contributing to the firmness of the skin. However, fibroblasts could also regulate skin pigmentation via paracrine signaling in humans (*34*–*36*). Notably, fibroblasts play a crucial role in regulating the pigmentation of palmoplantar skin, which is typically less pigmented and thicker than other parts of the body. Fibroblasts achieve this by secreting Dikkopf1 (DKK-1), which inhibits melanocyte growth and pigmentation (*37*). Conversely, fibroblasts can also promote melanogenesis by secreting neuregulin-1 (NGR-1) (*38*, *39*). Moreover, a coculture experiment involving fibroblasts with manipulated sFRP2 expression levels demonstrated that fibroblast-derived sFRP2 increased pigmentation in normal human melanocytes (*40*). Despite the differential effects of fibroblasts on pigment patterning, their specific roles in countershading have yet to be fully explored.

To date, ASIP is the only well-studied signaling pathway for countershading. The requirement for other genes or signaling pathways in countershading remains largely unknown. In this study, we aimed to elucidate the mechanism underlying the countershading of xanthophores in embryonic zebrafish. Specifically, we investigated the potential role of *col1a2^+^* fibroblasts in regulating this process. Our results demonstrate that the differential expression of *csf1a* and *csf1b* by fibroblasts and muscle progenitors plays a critical role in fine-tuning xanthophore countershading in embryonic zebrafish.

## RESULTS

### Countershading patterning of xanthophores is established in embryonic zebrafish

**Fig. 1.**
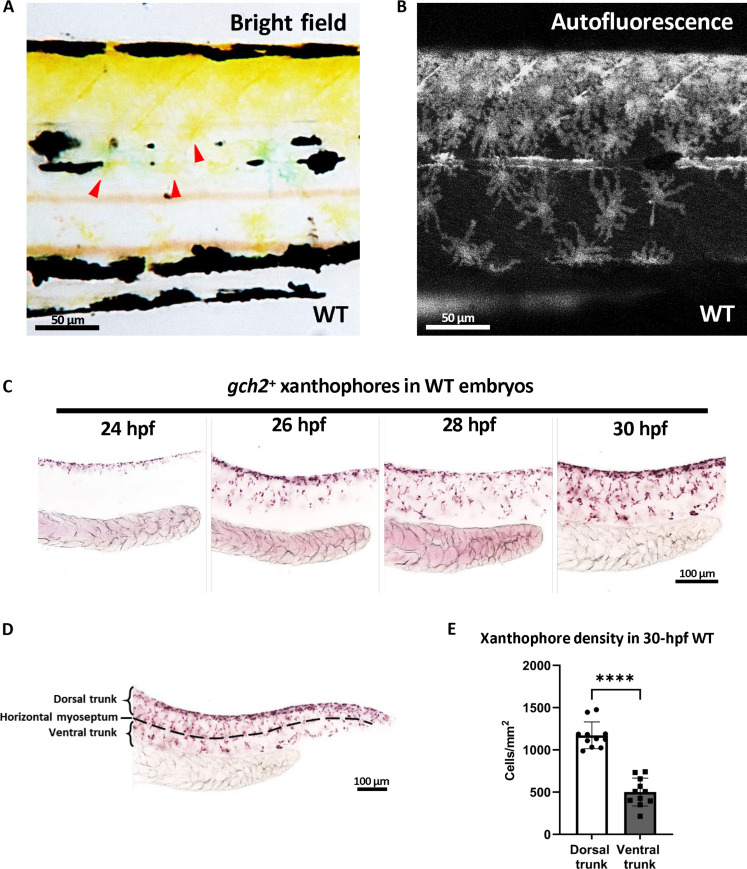
Xanthophore countershading in embryonic zebrafish. (**A** and **B**) Lateral views of 3-dpf zebrafish trunks. Red arrowheads indicated the differentiated xanthophores (A). Autofluorescent xanthophores on 3-dpf zebrafish trunks (B). More xanthophores reside at the dorsum than the ventrum. (**C**) Whole-mount in situ hybridization (WISH) staining at serial stages shows the *gch2*^+^ xanthoblasts migration begins at 24 hpf and finishes at 30 hpf. (**D**) Schematic diagram of the xanthoblasts/xanthophores counting region. The horizontal myoseptum is indicated by the black dotted line. (**E**) Quantification of *gch2*^+^ xanthophores density in the dorsal and ventral trunks, respectively. *n* = 9 for each group. Unpaired Student’s *t* tests were used to calculate the *P* value. Two-tailed *P* values are used. Error bars represent means ± SD. ****P < 0.0001. WT, wild type.

In embryonic zebrafish, xanthophores display a countershading pattern, with the dorsal trunk exhibiting a yellower color compared to the ventral part (Fig. 1A), which indicates that more xanthophores reside on the dorsal trunk than the ventral part. Using the autofluorescence of pteridine in xanthophores (*41*, *42*), we confirmed a higher abundance of autofluorescent xanthophores in the dorsal trunk compared to the ventral trunk (Fig. 1B). However, the detection of xanthophore autofluorescence was only possible after 40-hour postfertilization (hpf), by which time the early patterning of xanthophores was already established (Fig. 1C). To better understand the xanthophore patterning at early developmental stages, we used *gch2* as a specific marker to label early xanthophores (*32*, *43*). We found that xanthophores began to migrate from the dorsal part to the ventral part of the trunk at about 24 hpf and the patterning finished at 30 hpf (Fig. 1C). The horizontal myoseptum was used as the boundary to define the dorsal and ventral trunks of embryonic zebrafish (Fig. 1D). We observed a significantly higher xanthophore density in the dorsal trunk compared to the ventral trunk (Fig. 1E).

### Colony stimulating factors 1a and 1b are required for normal xanthophore countershading

**Fig. 2.**
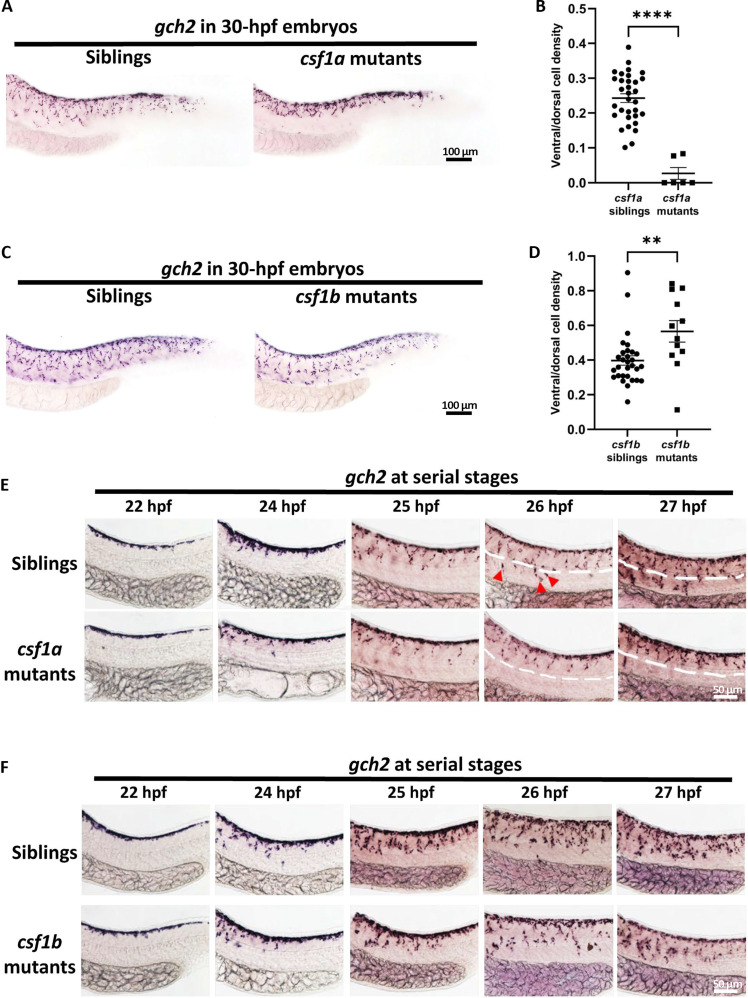
Xanthophore countershading is abnormal in *csf1a* and *csf1b* mutants. (**A** and **B**) Representative images (A) and quantification (B) of xanthophores in *csf1a* siblings (*n* = 32) and mutants (*n* = 6). Xanthophores are indicated by WISH staining of *gch2*. Unpaired Student’s *t* tests were used to calculate the *P* value. Two-tailed *P* values are used. Error bars represent means ± SD. ****P < 0.0001. (**C** and **D**) Representative images (C) and quantification (D) of xanthophores in *csf1b* siblings (*n* = 30) and mutants (*n* = 12). Xanthophores are indicated by WISH staining of *gch2*. Unpaired Student’s *t* tests were used to calculate the *P* value. Two-tailed *P* values are used. Error bars represent means ± SD. ns, *P* > 0.05; **P < 0.01. (**E**) WISH staining at serial stages shows that xanthoblasts in *csf1a* mutants fail to cross the horizontal myoseptum at about 26 hpf. The horizontal myoseptum is indicated by the white dotted line. (**F**) WISH staining at serial stages shows that xanthoblasts in *csf1b* mutants migrate normally toward the ventrum.

Csf1ra-Csf1a/Csf1b signaling is required for xanthophore development. However, their detailed roles on countershading remain unclear. To explore the roles of Csf1a and Csf1b in xanthophore countershading, we examined xanthophores in *csf1a* and *csf1b* mutants at 30 hpf, a stage when the countershading pattern of xanthophores has been established. In *csf1a* mutants, *gch2^+^* xanthophores accumulated on the dorsal trunk and showed limited migration across the horizontal myoseptum (Fig. 2A and fig. S1A). Quantification data revealed that the ventral/dorsal ratio of cell density drops drastically in *csf1a* mutants compared to their siblings (Fig. 2B). This decrease is attributed to a reduction in ventral xanthophore density, while dorsal xanthophore density remains unaffected in *csf1a* mutants (fig. S1A). In contrast, xanthophores in *csf1b* mutants successfully crossed the horizontal myoseptum (Fig. 2C). Intriguingly, a specific decrease of dorsal xanthophores led to significantly increased ventral/dorsal ratio of cell density in *csf1b* mutants comparing with the siblings (Fig. 2D and fig. S1B), suggesting altered countershading pattern. Previous report suggests that defective xanthophore patterning in juvenile zebrafish would affect the patterning of melanophores (*30*). Notably, we did not observe any differences in the number and distribution of melanophores between the mutant and sibling embryos (fig. S1, C to E). Similar to embryonic melanophores, no deficiency of embryonic iridoblasts distribution was observed in *csf1a* or *csf1b* mutants as exemplified by the *pnp4a* staining (fig. S1F). These results suggested that Csf1a and Csf1b play important and different roles in embryonic xanthophore patterning but not in melanocyte and iridophore patterning.

To further dissect the cellular defects of xanthophores in *csf1a* and *csf1b* mutants, we examined *gch2* expression from 22 hpf, before chromatoblasts migration, until 27 hpf, when the pattern of embryonic xanthophores was almost established. We found that xanthophores in *csf1a* mutants failed to migrate across the horizontal myoseptum at 26 hpf, whereas xanthophores in *csf1b* mutants exhibited normal migration (Fig. 2, E and F). To rule out the possibility of developmental delay as the cause of impaired migration in *csf1a* and *csf1b* mutants, we checked xanthophore pattern at 3-day postfertilization (dpf) and 11 dpf. We found that the restriction of xanthophores on the dorsal trunk of *csf1a* mutants remained at both 3 dpf and 11 dpf. And the decrease of dorsal xanthophore numbers in *csf1b* mutants also persist until 3 dpf and 11 dpf. Furthermore, xanthophores in *csf1a^−/−^csf1b^−/−^* mutants could not migrate ventrally until 11 dpf (fig. S2, A to C). It is worth noting that Csf1r signaling not only modulates cell migration but also regulates cell proliferation and differentiation (*32*). However, according to our data, the countershading pattern forms within a relatively short period of 6 hours (24 to 30 hpf), which is much shorter than the normal cell cycle (~24 hours), and we proposed that the major function of Csf1ra during this period is to direct the migration. Consistent with this notion, time-lapse imaging of transiently *TgBAC (csf1ra: eGFP)*–injected embryos showed most of migrating eGFP^+^ xanthophores did not proliferate during pattern formation (fig. S2D and movie S1).

### Ectopic expression of *csf1a* and *csf1b* recruits xanthophores to the central nervous system

**Fig. 3.**
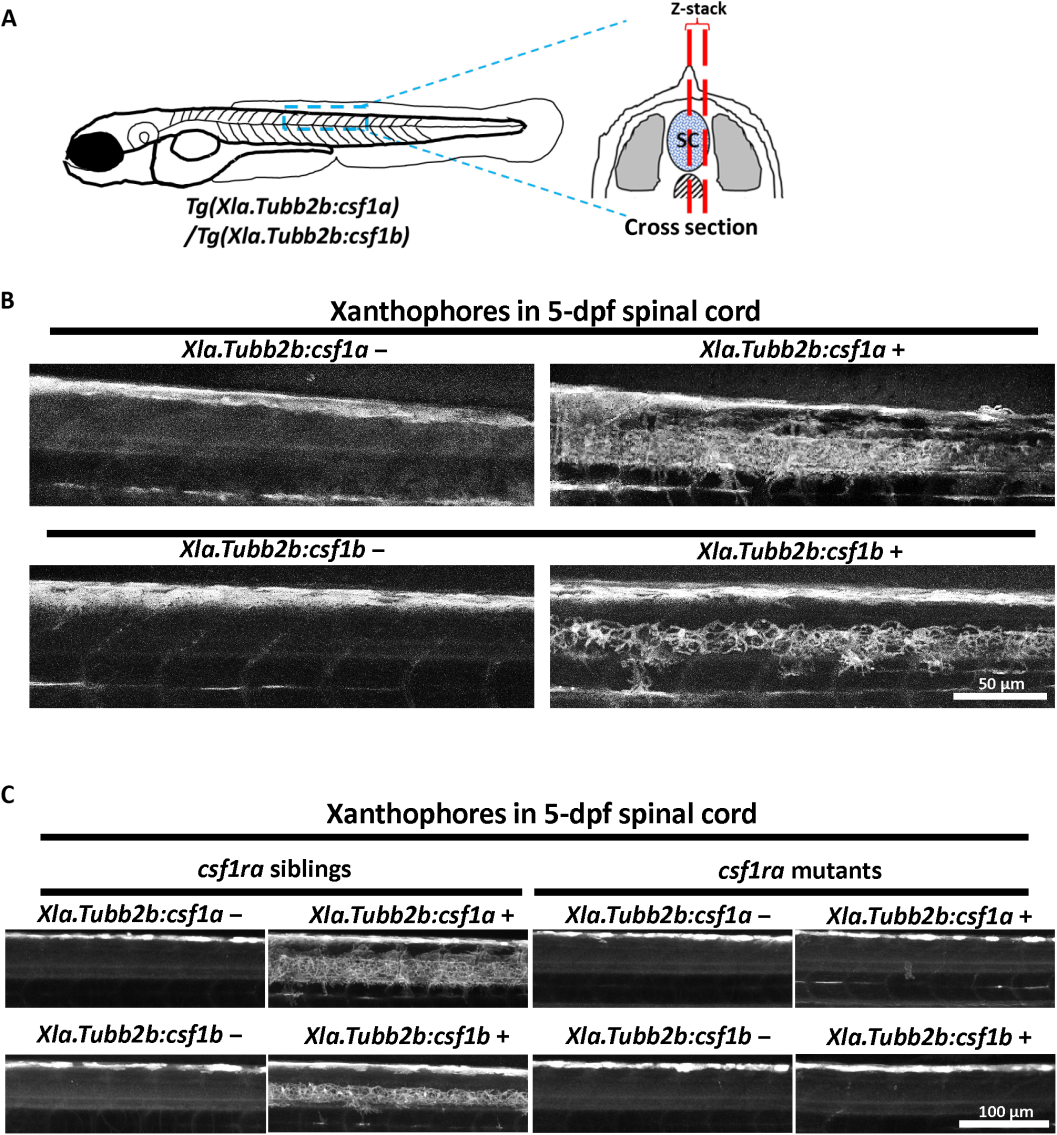
Csf1a and Csf1b are chemoattractants of xanthophores. (**A**) Schematic diagram of the imaging region. Blue dashed lines represent the imaging area at the dorsal trunk. Red dashed lines indicate the Z-stack range of confocal microscopy at spinal cord. SC, spinal cord. (**B**) Representative images of autofluorescent xanthophores at spinal cord region in 5-dpf larvae with (right) and without (left) *Tg(Xla.Tubb2b:csf1a)*/*Tg(Xla.Tubb2b:csf1b)*. Xanthophores are found in the spinal cord region of transgenic larvae. (**C**) Transgenic and nontransgenic *csf1ra* mutants or siblings are imaged at 5 dpf to observe autofluorescent xanthophore. Images show spinal cord regions in respective groups.

As ligands of Csf1ra, both Csf1a and Csf1b have the potential to recruit Csr1ra^+^ xanthophores by chemotaxis. To test this hypothesis in vivo, we used *Tg (Xla.Tubb:csf1a)* and *Tg(Xla.Tubb:csf1b)* to ectopically express Csf1a and Csf1b in the central nervous system (CNS), which lacks xanthophores/xanthoblasts under normal condition. If Csf1a and Csf1b function as chemoattractants, we would expect xanthophores to migrate into the CNS. As expected, we observed the appearance of xanthophores in the spinal cord region and their population peaked at 5 dpf (Fig. 3, A and B). To determine whether the attraction of xanthophores by Csf1a and Csf1b is dependent on the Csf1ra receptor, we crossed *csf1ra^j4e1^* (referred to as *csf1ra^−/−^* hereafter) with *Tg (Xla.Tubb:csf1a)* and *Tg (Xla.Tubb:csf1b)*. As expected, larvae lacking the Csf1ra receptor [*csf1ra^−/−^; Tg (Xla.Tubb:csf1a)* and *csf1ra^−/−^; Tg(Xla.Tubb:csf1b)*] did not exhibit xanthophores in the spinal cord region (Fig. 3C). Furthermore, similar to the phenotype observed in *csf1ra^−/−^* mutants, xanthophores in *csf1a^−/−^csf1b^−/−^* double mutants were confined to the dorsal edge of the trunk and failed to migrate ventrally at 28 hpf (fig. S2E). These findings strongly indicate that Csf1a and Csf1b act as chemoattractants for xanthophore migration via Csf1ra receptor.

### *csf1a* and *csf1b* are differentially expressed in the myotome/myoseptum during xanthophore pattern formation

**Fig. 4.**
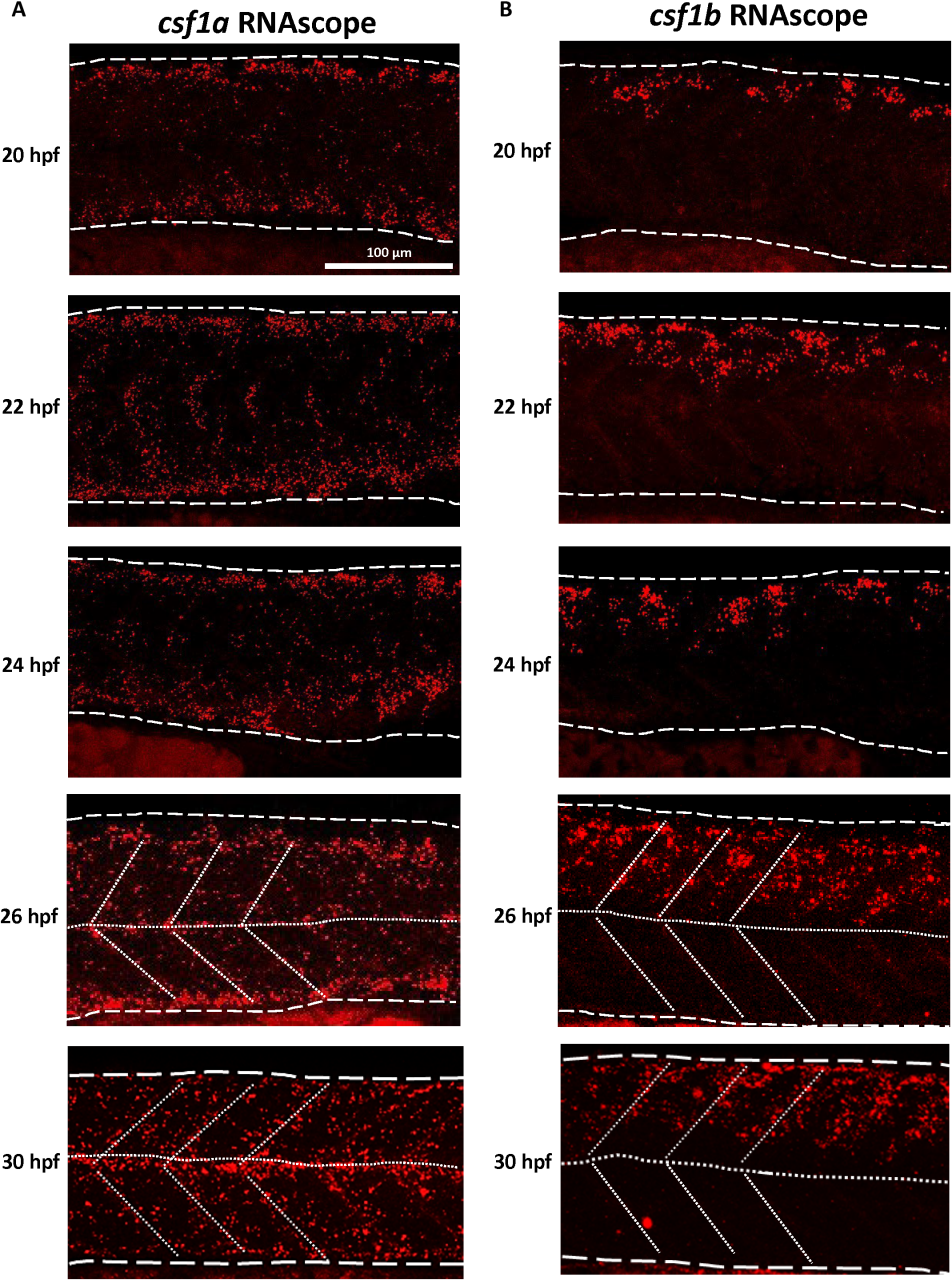
*csf1a* and *csf1b* are differentially expressed in the myotome and myoseptum during xanthophores pattern formation. (**A**) Representative images of RNAscope detecting *csf1a* RNA granules at 20, 22, 24, 26, and 30 hpf, respectively. Trunk region is indicated by the long dashed lines. Dotted lines indicate the horizontal and vertical myoseptum. (**B**) Representative images of RNAscope detecting *csf1b* RNA granules at 20, 22, 24, 26, and 30 hpf, respectively. Trunk region is indicated by the long dashed lines. Dotted lines indicate the horizontal and vertical myoseptum.

The robust chemoattraction observed in xanthophores strongly suggests that Csf1a and Csf1b play crucial roles in directing xanthophore migration and potentially influencing their patterning. A reasonable hypothesis is that *csf1a* and *csf1b* exhibit distinct expression patterns and work cooperatively to attract xanthophores, leading to the formation of the countershading pattern. If this is the case, then the distribution of xanthophores in *csf1a* or *csf1b* mutants should depict the expression of *csf1b* or *csf1a*, respectively: *csf1b* should only be expressed in the dorsal body, whereas *csf1a* should be expressed in both dorsal and ventral body. To test our hypothesis, we performed RNAscope staining to identify the expression pattern of *csf1a* and *csf1b* at 20, 22, 24, 26, and 30 hpf. As predicted, both *csf1a* and *csf1b* were expressed at 20 hpf before the xanthoblasts migration. *csf1a* at vertical myoseptum was detectable at 22 hpf, and it was expressed in both dorsal and ventral myotome/myoseptum at 30 hpf (Fig. 4A). In contrast, *csf1b* only showed expression in the dorsal myotome and vertical myoseptum from 20 to 30 hpf (Fig. 4B). We then concluded that the differential expression of *csf1a* and *csf1b* collectively governs the establishment of the xanthophore countershading pattern.

### *col1a2^+^* fibroblast and muscle progenitors are the primary source of Csf1a and Csf1b in embryonic zebrafish

**Fig. 5.**
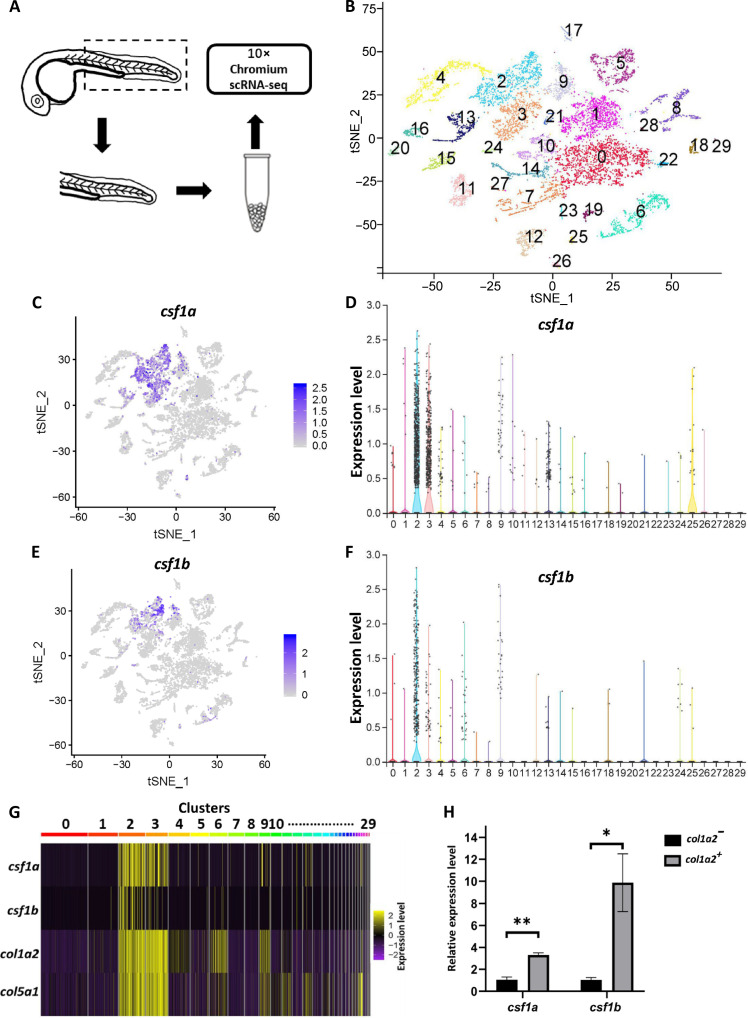
scRNA-seq reveals the sources of Csf1a and Csf1b in embryonic zebrafish. (**A**) Scheme of the sample preparation workflow. Trunks of 27- to 28-hpf embryos were dissected and dissociated into single cells. (**B**) Clustering of the single-cell transcriptome results in 30 clusters (label with different colors and no. 0 to 29). (**C** and **D**) Expression analysis of *csf1a* in t-SNE plot (C) and violin plot (D). The results showed that *csf1a* expression is largely restricted to cells in clusters 2 and 3. (**E** and **F**) Expression analysis of *csf1b* in t-SNE plot (E) and violin plot (F). The results showed that *csf1b* expression is largely restricted to cells in cluster 2. (**G**) Heatmap of normalized differentially expressed genes: *csf1a, csf1b, col1a2,* and *col5a1* in all 30 clusters. (**H**) Relative expression levels of *csf1a* and *csf1b* in Col1a2^+^ and Col1a2^−^ cells, respectively. Col1a2^+^ and Col1a2^−^ cells (*n* = 50,000, respectively) were sorted from Tg (*col1a2: Gal4; UAS: NTR-mCherry*). Data represented four independent experiments. Error bars represent mean ± SD. Unpaired, two-tailed *t* test was performed to determine significance. *P < 0.05 and **P < 0.01.

Previous studies have indicated that iridophores serve as the source of Csf1a and Csf1b in juvenile zebrafish (*30*). However, the expression pattern of *csf1a* and *csf1b* in embryonic zebrafish does not follow the distribution pattern of iridophores (Fig. 4 and fig. S1F). We further examined xanthophores in *mpv17* mutants in which iridophores are severely defective (*44*, *45*). The xanthophore pattern was normal in *mpv17* mutants, suggesting that iridophores were dispensable for embryonic xanthophore patterning (figs. S3, A and B). To identify the source of Csf1a and Csf1b, we conducted single-cell RNA sequencing (scRNA-seq). Trunks from 28 hpf embryos were collected and subjected to 10X Genomics scRNA-seq (Fig. 5A). Subsequently, 10797 single-cell transcriptomes were classified into 30 clusters (Fig. 5B). According to the t-distributed stochastic neighbor embedding (t-SNE) clustering results, *csf1a* was mainly expressed in cluster 2 and cluster 3, whereas *csf1b* was primarily expressed in cluster 2 (Fig. 5, C to F). Previously, Sharma *et al.* (*46*, *47*) generated a transgenic line *Tg(col1a2:Gal4;UAS:NTR-mCherry)* (referred to as *col1a2^NTR-mCherry^* hereafter) to label *col1a2^+^* fibroblast and muscle progenitors in zebrafish. We noticed that, like *csf1a* and *csf1b*, *col1a2* was also specifically enriched in clusters 2 and 3 (Fig. 5G). Further analysis of differentially expressed genes in these clusters helped us identify some signature genes. Both clusters 2 and 3 expressed pan-fibroblast markers such as *col5a1*, *pdgfra*, and *tgfbi* (Fig. 5G and fig. S4). Cluster 2 specifically enriched genes such as *myf5*, *pax3a*, and *pax7a* which are typical markers of muscle progenitors (*48*–*52*). In contrast, cluster 3 cells express *nkx3-1*, *pax1a*, and *pax9* which are markers of fibroblast precursors (fig. S4) (*53*, *54*). We therefore identify cells in cluster 2 as *csf1a^high^csf1b^high^* muscle progenitors and cells in cluster 3 as *csf1a^high^csf1b^low^* fibroblast progenitors. Using the *col1a2^NTR-mCherry^* transgenic line, we collected mCherry^+^ and mCherry^−^ cells by fluorescence-activated cell sorting (FACS) at 28 hpf. The expression level of *csf1a* and *csf1b* was examined in these two populations via real-time polymerase chain reaction (PCR). We found that both *csf1a* and *csf1b* were enriched in the mCherry^+^ population (Fig. 5H). Together, these data suggested that *col1a2^+^* fibroblast and muscle progenitors were the primary sources of *csf1a* and *csf1b* in embryonic zebrafish.

### Ablation of *col1a2^+^* fibroblast and muscle progenitors disrupts the xanthophore patterning in embryonic zebrafish

To test whether *col1a2^+^* fibroblast and muscle progenitors are essential for xanthophore patterning in embryonic zebrafish, we ablated *col1a2^+^* cells with the nitroreductase (NTR)–based system. The NTR enzyme could convert the harmless prodrug metronidazole (MTZ) into a cytotoxic molecule, inducing cell death specifically in NTR-expressing cells (*55*, *56*). In our experiments, we leveraged ronidazole (ROZ), an analog of MTZ with higher cell ablation efficiency, to deplete *col1a2^+^* fibroblast and muscle progenitors (*57*).

**Fig. 6.**
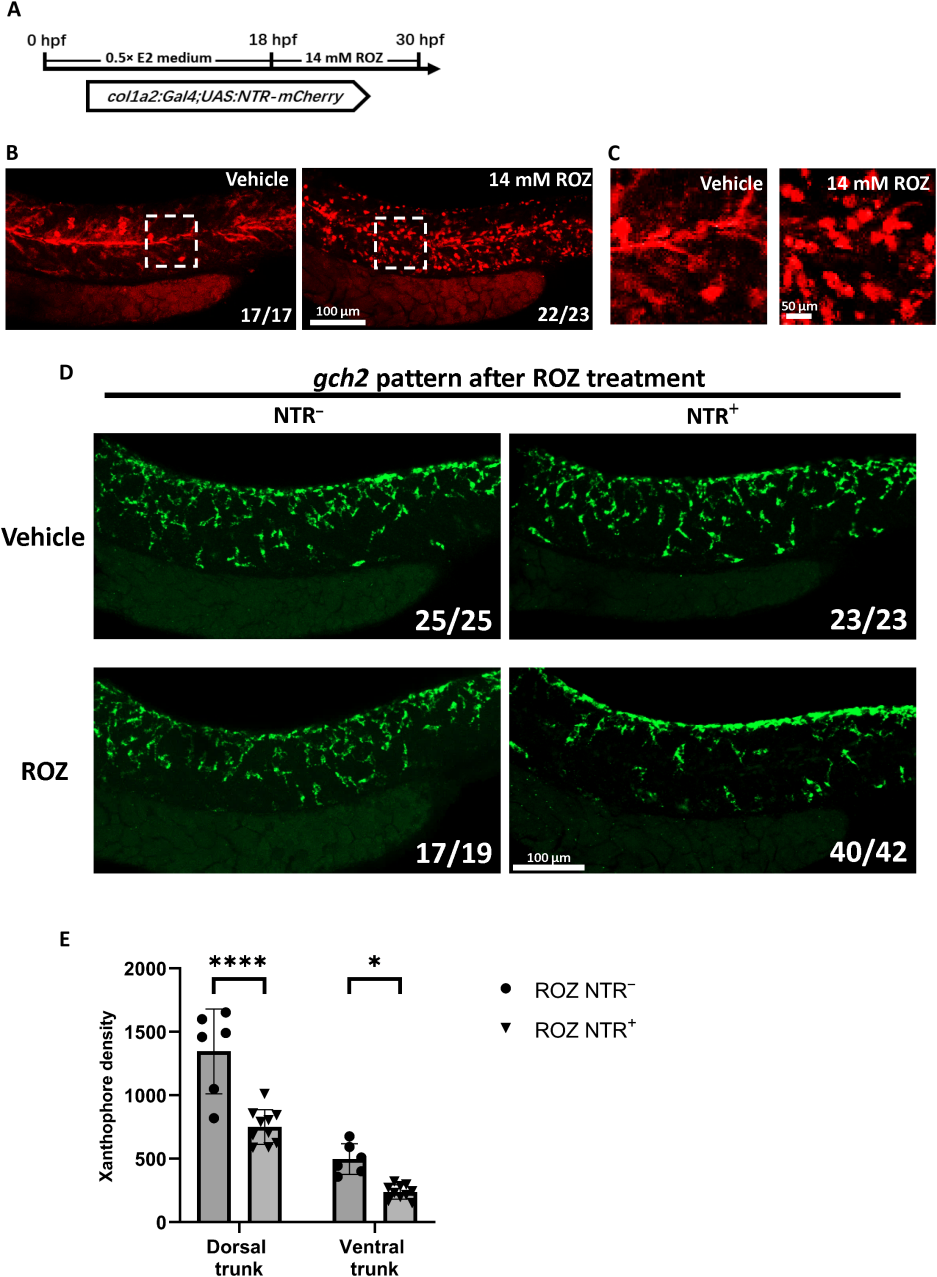
Ablation of *col1a2^+^* fibroblast and muscle progenitors disrupts the xanthophore countershading in embryonic zebrafish. (**A**) Scheme of ROZ treatment workflow. (**B**) Representative images of *col1a2^+^* cells after ROZ treatment. Data are representative of the majority of embryos analyzed (proportion indicated lower right of each panel). *col1a2^+^* cells are indicated by *Tg (col1a2: Gal4;UAS:NTR-mCherry)*(red). (**C**) Magnified images of white dashed box in (B). (**D**) Representative images of *gch2* fluorescent in situ hybridization (FISH) after ROZ treatment. Data are representative of the majority of embryos analyzed (proportion indicated lower right of each panel). (**E**) Quantification of xanthophores density in ROZ-treated embryos with NTR transgene (*n* = 10) and without NTR transgene (*n* = 6). Xanthophores are indicated by FISH staining of gch2. Significances were calculated using two-way analysis of variance (ANOVA) followed by Sidak’s multiple comparisons test and Tukey’s multiple comparisons test. Error bars represent means ± SD. *P < 0.05 and ****P < 0.0001.

We treated *col1a2^NTR-mCherry^* embryos with 14 mM ROZ from 18 hpf and assessed the cell ablation efficiency as well as the expression of *csf1a* and *csf1b* at 30 hpf (Fig. 6A). After treatment, we observed that the *col1a2^+^* cells exhibited shrinkage and a rounded morphology, indicating cell death (Fig. 6, B and C). Subsequently, we investigated whether the ablation of *col1a2^+^* cells resulted in reduced expression of *csf1a* and *csf1b*. As anticipated, both *csf1a* and *csf1b* signals were decreased in ROZ-treated *col1a2^NTR-mCherry^* transgenic embryos (NTR^+^) comparing to nontransgenic control embryos (NTR^−^) (fig. S5, A and B). Consequently, we found that xanthophores in the ROZ-treated transgenic embryos exhibited significantly impaired ventral migration when compared to those in the nontransgenic embryos (Fig. 6, D and E). Together, our results suggested that *col1a2^+^* fibroblast and muscle progenitors were essential for xanthophore countershading by differentially expressing *csf1a* and *csf1b* in embryonic zebrafish.

## DISCUSSION

In this study, we elucidate a mechanism governing countershading, distinct from the well-established ASIP signaling known to regulate countershading in both mammals and fishes (*2*, *27*). We found that the *col1a2^+^* fibroblast and muscle progenitors, which are widely distributed at the myotome and myoseptum regions, differentially express Csf1a and Csf1b, the natural ligands of Csf1ra, to attract xanthoblasts/xanthophores to the proper regions and form the countershading pattern. This countershading of xanthophores is largely overlooked previously since the xanthophore in embryonic zebrafish is thought to be sparsely distributed (*30*). Unlike other well-studied countershading patterns in mammals or fishes, the regulation of this countershading of xanthophores does not rely on the control of pigmentation or maturation of pigment cells (*27*, *29*). Instead, a proper guidance of chemoattractant is necessary. Therefore, we propose Csf1a/1b-Csf1ra to be a second signaling, in addition to the ASIP signaling, for countershading. Csf1ra, an ortholog of CSF1R, is expressed on xanthophores but is unrelated to the synthesis of yellow pteridine pigments. CSF1R plays a crucial role in cell motility, migration, proliferation, and differentiation (*32*). In contrast, ASIP signaling influences pigmentation by regulating melanin type and melanocyte maturation via MC1R, crucial for eumelanin synthesis. We propose that during countershading formation, Csf1ra primarily guides xanthophore migration rather than proliferation or differentiation. Furthermore, ectopic expression of *csf1a* and *csf1b* resulted in the recruitment of xanthophores to the spinal cord. However, considering the development of adult pigment precursors during embryonic stages, with some being identified in association with peripheral nerves (*58*, *59*), we could not rule out the possibility that some of the xanthophores we found in the spinal cord originated from these precursors. Other potential functions of Csf1r on the formation of countershading remain to be explored, and it may influence the countershading pattern in later stage by proliferation and differentiation to help setup adult pigment patterning. We noticed that ectopically expressed Csf1a led to more xanthophores in the spinal cord than Csf1b did. This result suggests that Csf1a may have stronger chemoattraction ability than Csf1b. Alternatively, Csf1a may better promote the proliferation of xanthophores than Csf1b.The protein similarity between Csf1a and Csf1b is about 49%, and we believe that the differences in affinity and positioning upon receptor binding contribute to variations in downstream signal activation. This similar phenomenon can be gleaned from homologous signaling like CSF1/IL34 ligands and CSF1R receptor in mammals (*60*, *61*). Last, we could not exclude the possibility that the variation of insertion sites and copy numbers of transgenes leads to different amount of chemokine in the spinal cord. Countershading is a universal pigment pattern and is conserved among various species. Whether the Csf1a/1b-Csf1ra signaling plays roles in countershading of other species warrants further study.

Previous studies reported Csf1a and Csf1b expression in iridophores of juvenile and adult zebrafish (*30*). Csf1b was also identified in hypodermis of juvenile/adult zebrafish (*62*). Different from the juvenile and adult stage, we found that embryonic Csf1a and Csf1b were differentially expressed in *col1a2^+^* fibroblast and muscle progenitors instead. Zebrafish displays different pigment patterns including the embryonic/early larval pattern and the adult pattern during their development. The adult pattern with characteristic stripes could be seen at the end of metamorphosis when a “juvenile pattern” is formed with one light-colored interstripe bordered by two dark-colored stripes (*30*, *63*). The stipe formation required organized cell-cell interactions, and the dynamic of these cells displays properties of self-activation or lateral inhibition which could refer to as a reaction-diffusion system—a “Turing mechanism” (*64*, *65*). The *csf1a* expressed by interstripe iridophores is reported to promote xanthophore development and stripe formation (*30*, *31*). However, we found that the xanthoblasts/xanthophore pattern of embryonic zebrafish was not affected in iridophore mutants. Adult *csf1b* mutants have recently been noted to exhibit a reduction in dorsal xanthophores (*62*), a phenotype akin to what we observed in embryonic *csf1b* mutants. It is possible that Csf1b executes similar function in both stages. However, it is crucial to recognize the distinct origins of Csf1b between the embryonic stage (Col1a2^+^ fibroblasts and muscle progenitors) and adulthood (hypodermis). The altered origin of Csf1b may be attributed to the substantial changes in cellular and tissue environments from the embryonic stage to adulthood, including structural modifications in zebrafish skin. It would be intriguing to explore whether the Csf1b-expressing hypodermis in adults shares a common origin with embryonic Col1a2^+^ fibroblasts and muscle progenitors. On the other hand, *csf1ra* mutant, but not single *csf1a* or *csf1b* mutant, completely disrupted the adult stripe (*62*). Given the fact that both *csf1a* and *csf1b* are expressed by iridophore in adult zebrafish and iridophore plays an essential role in adult stripe pattern formation (*30*). These findings suggest that *csf1a* and *csf1b* play redundant roles in adult iridophores. Together, these results indicate different cellular and molecular machineries of xanthophore patterning between embryonic and adult zebrafish. *col1a2^−/−^* adult fishes are viable and display disturbed stripe pattern in the skin compared to the heterozygous and wild-type fishes (*66*). This observation indicates that Col1a2 also plays roles in adult pigment patterning, but the mechanism by which *col1a2^+^* cells regulate pigment patterning in adult zebrafish remains to be explored.

Our results also addressed special functions of the *col1a2^+^* fibroblast and muscle progenitors in zebrafish. The inter- and intra-organ heterogeneity among the fibroblasts has been found recently (*67*). Fibroblast subtypes express diverse extracellular matrix components in distinct anatomical niches and exhibit varied roles in pigmentation. For instance, Dikkopf1 expression in palmoplantar skin fibroblasts inhibits melanogenesis in melanocytes, while clusterin and pleiotrophin have shown melanogenesis inhibition in vitro (*68*, *69*). In contrast, fibroblasts could promote melanin synthesis by expressing molecules such as NRG1 in dark skin (*38*, *39*), KGF, and Sema7a after UV exposure (*70*–*72*). This functional heterogeneity could attribute to the heterogeneity of fibroblast populations. Fibroblasts are able to recruit circulating leucocytes and activate endothelium under inflammatory condition (*73*–*75*). Furthermore, cancer-associated fibroblasts were reported to promote metastasis of cancer cells (*76*, *77*). These reports suggest that fibroblasts could modulate cell migration.

In our study, we found that *col1a2*^+^ fibroblasts and muscle progenitors express *csf1a* and *csf1b*, attracting xanthoblasts/xanthophores. *csf1a* and *csf1b* show distinct expression patterns in these cells. Our scRNA-seq results confirmed this differential expression. We suspect that the distinct microenvironments in which *col1a2^+^* cells reside could lead to this different expression patterns of *csf1a* and *csf1b*. The dorsal trunk region is reported to specifically express molecules such as Zic1 and Zic4 to regulate the asymmetrical morphology of the trunk of medaka, like fin morphology and pigmentation (*78*). In addition, Wnt signals are expressed at the dorsal region of neural tube to induce myogenesis. These dorsal-specific signals could form a special niche for fibroblast at the dorsum and possibly affect their properties. A recent study in zebrafish showed that sclerotome, a portion of the somite, could generate different fibroblast subtypes by sensing different local signals (*54*). It is conceivable that the subpopulations of fibroblast and muscle progenitors expressing Csf1a and Csf1b may originate from distinct sources. Studies in mice have suggested that fibroblasts within the same tissue might derive from different embryonic origins (*79*–*81*). To test this possibility, lineage tracing studies are required. The cellular origins and biology significance of fibroblast subpopulations are largely unclarified. Our study provides a model for further investigating the functional heterogeneity of fibroblasts in zebrafish.

Together, we have identified Col1a2^+^ fibroblasts and muscle precursor cells that refine embryonic zebrafish countershading by differentially expressing *csf1a* and *csf1b.* Further exploration of the reasons for the heterogeneity in *csf1a* and *csf1b* expression in different tissue of embryonic and adult zebrafish will contribute to a better understanding of the formation mechanism and biological significance of xanthophore countershading. This, in turn, will provide valuable mechanistic insights for embryonic pattern formation.

## MATERIALS AND METHODS

### Zebrafish husbandry and lines used

All zebrafish lines were maintained according to the standard protocol (*82*). Zebrafish were reared at 28.5°C in a 14-hour light and 10-hour dark cycle. After natural spawning, embryos were collected and reared in 0.5× E2 medium containing 0.00005% methylene blue (egg water) at 28.5°C. To avoid pigmentation, embryos were changed to 0.003% *N*-phenylthiourea (P7629, Sigma-Aldrich) in egg water at 1 dpf. AB wild-type, *roy* (*45*), *csf1ra^j4e1^* (*32*), *Tg(col1a2^NTR-mCherry^)* short for *Tg(col1a2:Gal4;UAS:NTR-mCherry)* (*47*), *Tg (Xla.Tubb:csf1a)^hkz17Tg^*, *Tg(Xla.Tubb:csf1b)^hkz18Tg^*, *csf1a^hkz9^*, and *csf1b^hkz10^* (*33*) are used in this study. All animal procedures and experiments were conducted according to the guidelines of the South China University of Technology (SCUT). All the animal experiments were approved by the Animal Ethics Committee of SCUT.

### WISH, FISH, and RNAscope in situ analysis

Antisense digoxigenin (DIG)–labeled *gch2* probe was synthesized in vitro as described (*83*) and diluted with hybridization buffer to 1 ng/μl. Conventional and fluorescent whole-mount in situ hybridization (WISH and FISH) was performed according to a protocol modified from the previous study. Briefly, embryos were anesthetized, fixed in 4% paraformaldehyde (PFA), and permeabilized with 100% methanol. After rehydration with 1× Phosphate Buffered Saline with Tween 20 (PBST), embryos were furthered permeabilized using proteinase K (10 μg/ml; EO0492, Thermo Fisher Scientific), then refixed with 4% PFA, and prehybridized in hybridization buffer for 2 to 3 hours. Next, embryos were changed to hybridization buffer containing DIG-labeled RNA probe and incubated at 65°C overnight. In the next day, embryos were applied to a series of stringent washing using different concentrations of SSC buffer and transferred into maleic acid buffer (MAB). After the MAB washing, the embryos were incubated with the blocking buffer (2% blocking reagent, 11096176001 Roche) at room temperature for 1 hour and then with anti–DIG–Alkaline phosphatase (AP) (11093274910, Roche) (1:5000 dilution in blocking buffer) or anti–DIG-Peroxidase (POD) (11207733910, Roche) (1:2000 dilution in blocking buffer). After washing several times with Maleic Acid Buffer with Tween 20 (MABT), embryos were stained with bromochloroindolyl phosphate–nitro blue tetrazolium solution (11697471001, Roche) or the TSA Plus Fluorescein System (NEL741001KT, Akoya Biosciences). RNAscope was conducted with probes csf1a (catalog no. 564771, ACD Bio), csf1b (catalog no. 809061, ACD Bio), and negative control probe (catalog no. 310043, ACD Bio). RNAscope procedure was performed following the manufacturer’s instructions (MK 50-016, 323100-USM, ACD Bio). Images of WISH were mounted in 70% glycerol and captured by a Zeiss Axio Zoom.V16 stereo microscope. FISH and RNAscope were imaged by a Zeiss LSM800 confocal microscope.

### Cell isolation, flow cytometry, cDNA synthesis, and qPCR

Trunks were dissected from 30-hpf embryos and digested with 0.25% trypsin-EDTA (25200072, Gibco) at 30°C for 30 to 40 min. The digestion reaction was stopped by addition of 10 mM CaCl_2_ and 10% fetal bovine serum (FBS). The dissociated cells were then washed with ice-cold 1% bovine serum albumin (BSA)/phosphate-buffered saline (PBS) buffer, resuspended, and passed through a 40-mm cell strainer (352340, BD Falcon). The FACS analysis was performed on MoFlo Astrios EQ (Beckman Coulter). A total of 50,000 mCherry^−^ cells and 50,000 mCherry^+^ cells were directly sorted into TRIzol reagent (15596018, Invitrogen). Then, RNA was extracted and reverse-transcribed with Super-Script IV Reverse Transcriptase (18090050, Invitrogen). Real-time quantitative PCR (qPCR) was performed to examine the transcripts corresponding to the coding region of *csf1a* and *csf1b*. Reference gene *eef1a1l1* is used for internal control. Primers for qPCR are listed in table S2.

### Embryo dissociation

Trunks were dissected from 28-hpf embryos and digested with 0.25% trypsin-EDTA (25200072, Gibco) at 30°C for 30 to 40 min. The digestion reaction was stopped by addition of 10 mM CaCl_2_ and 10% FBS. The dissociated cells were then washed with ice-cold 1% BSA/PBS buffer, resuspended, and passed through a 40-mm cell strainer (352340, BD Falcon). The percentage of viable cells was measured on a cell count plate after staining with 0.4% trypan blue (T6146, Sigma-Aldrich).

### 10× Chromium scRNA-seq library construction

Cellular suspensions were loaded on a 10X Genomics GemCode single-cell instrument that generates single-cell Gel Bead-In-EMulsion (GEMs). Libraries were generated and sequenced from the cDNAs with Chromium Next GEM Single Cell 3′ Reagent Kits v3.1. Upon dissolution of the Gel Bead in a GEM, primers containing (i) an Illumina R1 sequence (read 1 sequencing primer), (ii) a 16-nt 10× barcode, (iii) a 10-nt unique molecular identifier (UMI), and (iv) a poly-dT primer sequence were released and mixed with cell lysate and Master Mix. Barcoded, full-length cDNAs were then reverse-transcribed from poly-adenylated mRNA. Silane magnetic beads were used to remove leftover biochemical reagents and primers from the post-GEM reaction mixture. Full-length, barcoded cDNAs were then amplified by PCR to generate sufficient mass for library construction. R1 were added to the molecules during GEM incubation. P5, P7, a sample index, and R2 were added during library construction via end repair, A-tailing, adaptor ligation, and PCR. The final libraries contained the P5 and P7 primers used in Illumina bridge amplification (*84*).

### scRNA-seq read alignment and quantification

Raw reads were aligned to version 11 of the zebrafish genome (GRCz11) using the standard Cell Ranger pipeline from 10X Genomics (version 3.1.0). Briefly, reads with low-quality barcodes and UMIs were filtered out and then mapped to the reference genome. Reads uniquely mapped to the transcriptome and intersecting an exon at least 50% were considered for UMI counting. Before quantification, the UMI sequences would be corrected for sequencing errors, and valid barcodes were identified on the basis of the EmptyDrops method (*85*). Resulting barcodes were used to generate a UMI matrix for further analysis. The scRNA-seq data were deposited in the National Center for Biotechnology Information (NCBI) SRA database: PRJNA981358.

### Quality control and filtering

The UMI matrix was imported to Seurat (*86*) version 3.1.1 for downstream analysis. Cells with unusually high number of UMIs (≥8000) or mitochondrial gene percent (≥10%) were filtered out. We also excluded cells with less than 500 or more than 4000 genes detected. In addition, doublet GEMs also should be filtered out. It was achieved by using the tool DoubletFinder (v2.0.3) by the generation of artificial doublets, using the principal component (PC) distance to find each cell’s proportion of artificial *k*-nearest neighbors and ranking them according to the expected number of doublets (*84*). After filtering, we detected 355,595 genes in total and 10,797 cells.

### Dimensional reduction and cell clustering

Integrated expression matrix is scaled and performed on principal components analysis (PCA) for dimensional reduction. Then, we implemented a resampling test inspired by the JackStraw procedure. We randomly permuted a subset of the data (1% by default) and rerun PCA, constructing a “null distribution” of gene scores, and repeated this procedure. We identified “significant” PCs as those who have a strong enrichment of low *P* value genes for downstream clustering and dimensional reduction. Seurat implements a graph-based clustering approach. Distances between the cells were calculated on the basis of previously identified PCs. Briefly, Seurat embed cells in a shared-nearest neighbor (SNN) graph, with edges drawn between cells via similar gene expression patterns. To partition this graph into highly interconnected quasi-cliques or communities, we first constructed the SNN graph based on the Euclidean distance in PCA space and refined the edge weights between any two cells based on the shared overlap in their local neighborhoods (Jaccard distance). We then cluster cells using the Louvain method to maximize modularity. For visualization of clusters, t-SNE were generated using the same PCs (*87*).

### Differentially expressed genes analysis

Expression value of each gene in given cluster was compared against the rest of cells using Wilcoxon rank sum test. Significant up-regulated genes were identified using a number of criteria. First, genes had to be at least 1.28-fold overexpressed in the target cluster. Second, genes had to be expressed in more than 25% of the cells belonging to the target cluster. Third, a *P* value is less than 0.05 (*88*).

### ROZ treatment

Eighteen–hour postfertilization embryos were dechorioned and transferred to a new petri dish. ROZ (14 mM; R7635, Sigma-Aldrich) was freshly prepared in E2 medium containing 0.003% *N*-phenylthiourea. Embryos were immersed in the 14 mM ROZ in dark at 28.5°C and developed until 30 to 31 hpf. Control embryos were reared in standard E2 medium containing 0.003% *N*-phenylthiourea in dark at 28.5°C.

### Quantification and statistical analysis

Quantification of xanthophore density involved manual cell counting within the designated counting region, as illustrated in Fig. 1D. The area for calculation was determined using Carl Zeiss ZEN Blue. The density of xanthophores (cells per square millimeter) was calculated by dividing the total cell count by the area of the counting region (square millimeters). All presented data represented at least three independent experiments. All the statistical analyses were performed using GraphPad Prism version 9.0.2. Unpaired Student’s *t* tests were used to calculate the *P* value for pairwise comparisons. Two-tailed *P* values are used for all *t* tests. For multiple comparisons, significances were calculated using one-way analysis of variance (ANOVA) followed by Dunnett’s multiple comparisons test or two-way ANOVA followed by Sidak’s multiple comparisons test and Tukey’s multiple comparisons test.
