# Supplementary material for: col1a2+ fibroblasts/muscle progenitors finetune xanthophore countershading by differentially expressing csf1a/1b in embryonic zebrafish
Source: Sci Adv. 2024 Apr 5;10(14):eadj9637. doi: 10.1126/sciadv.adj9637 (PMC10997200; doi:10.1126/sciadv.adj9637)
Supplement: Supplementary file 1 — Figs. S1 to S5 Tables S1 and S2 Legend for movie S1 [file sciadv.adj9637_sm.pdf]

Science Advances
AAAS

Supplementary Materials for

col1a2^{+} fibroblasts/muscle progenitors finetune xanthophore countershading by differentially expressing *csf1a/1b* in embryonic zebrafish

Jiahao Chen *et al.*

Corresponding author: Jin Xu, xujin@scut.edu.cn

Sci. Adv. **10**, eadj9637 (2024)
DOI: 10.1126/sciadv.adj9637

The PDF file includes:

Figs. S1 to S5
Tables S1 and S2
Legend for movie S1

Other Supplementary Material for this manuscript includes the following:

Movie S1

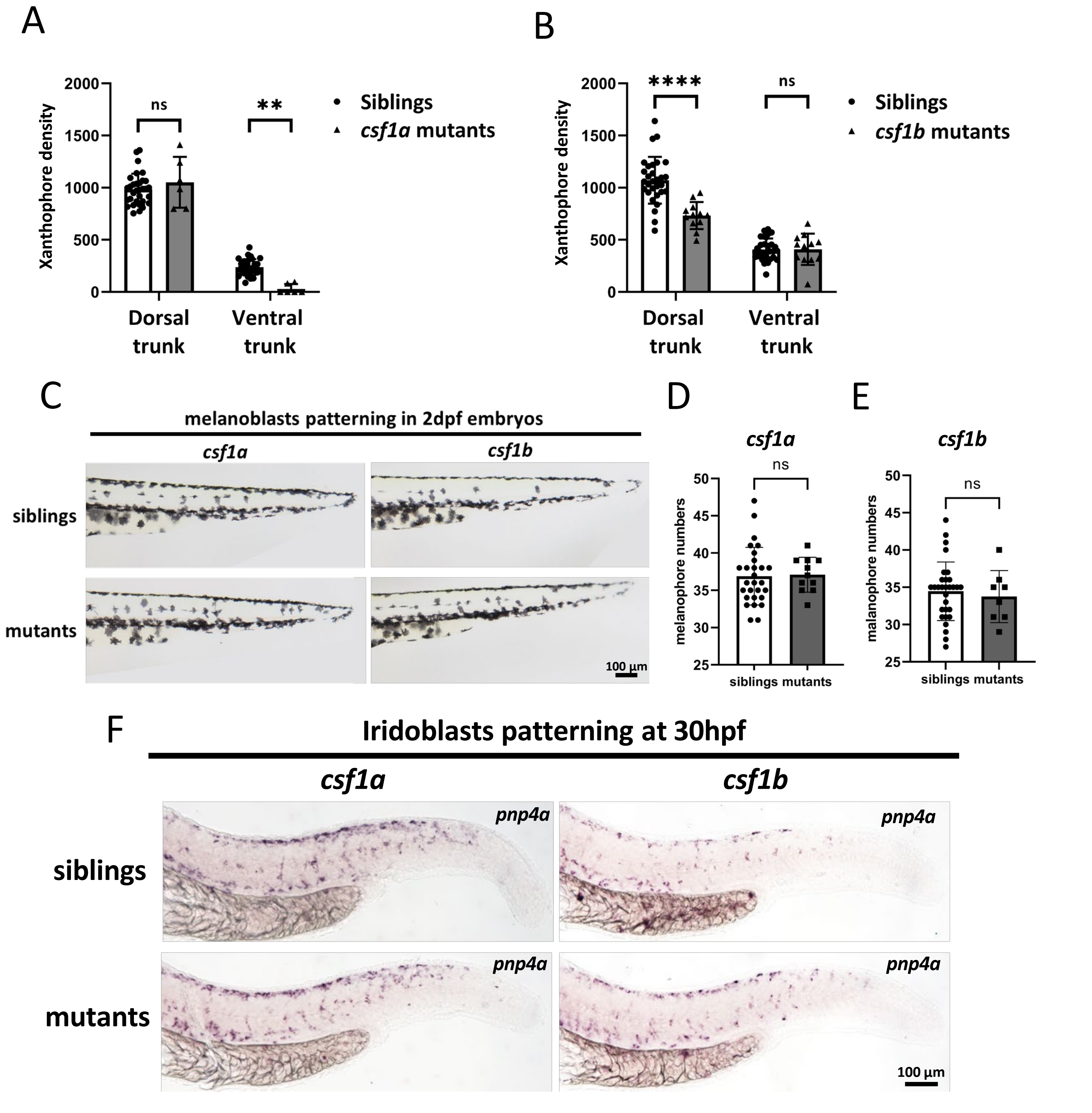

Figure S1. Melanophores and iridophores are not affected in csf1a and csf1b mutants, related to Figure 2

(A and B) Quantification of xanthophores density in csf1a siblings(n=32) and mutants(n=6) (A), in csf1b siblings(n=30) and mutants(n=12) (B). Xanthophores are indicated by WISH staining of gch2. Error bars represent means ± SD. ns, p > 0.05; **p<0.01, ****p<0.0001.

(C-E) Representative images(C) and quantification of melanoblasts numbers in the trunk of 48-hpf csf1a mutants(n=11), csf1a siblings(n=28) (D), csf1b mutants(n=8) and csf1b siblings(n=30) (E). Error bars represent means ± SD. ns, p>0.05.

(F) Representative images of iridoblast pattern in 30-hpf csf1a mutants, csf1a siblings, csf1b mutants and csf1b siblings. Iridoblasts are indicated by WISH staining of pnp4a.

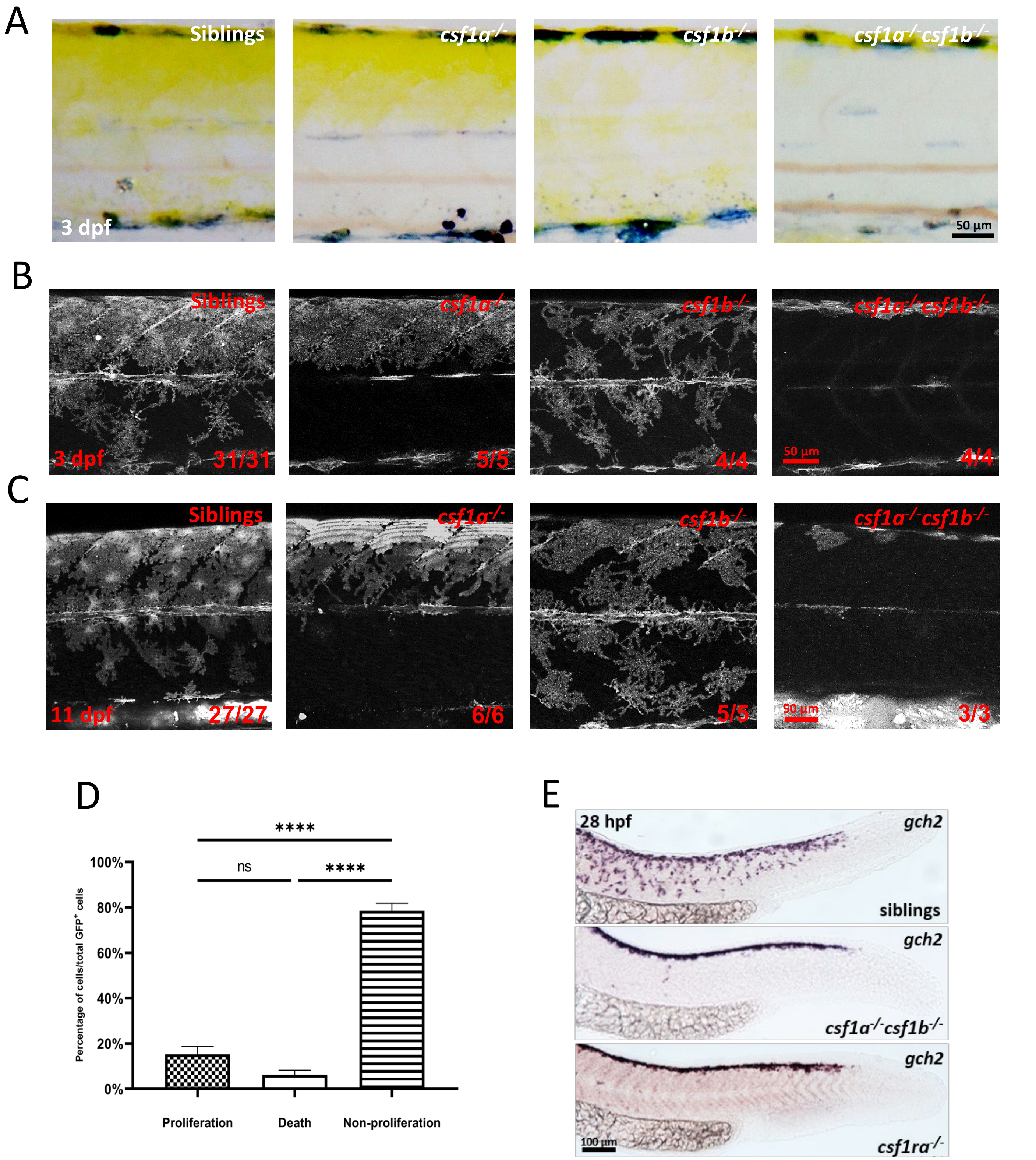

Figure S2. countershading pattern in *csf1a*$^{-/-}$, *csf1b*$^{-/-}$ mutants and *csf1ra* mutants. Related to Figure 2 and 3

(A) Brightfield image of xanthophore pattern in 3 dpf *csf1a*$^{-/-}$,*csf1b*$^{-/-}$ and *csf1a*$^{-/-}$*csf1b*$^{-/-}$ mutants and siblings.

(B) Fluorescent xanthophore pattern of 3 dpf *csf1a*$^{-/-}$,*csf1b*$^{-/-}$ and *csf1a*$^{-/-}$*csf1b*$^{-/-}$ mutants and siblings.

(C) Fluorescent xanthophore pattern of 11 dpf *csf1a*$^{-/-}$,*csf1b*$^{-/-}$ and *csf1a*$^{-/-}$*csf1b*$^{-/-}$ mutants and siblings.

(D) Quantification of eGFP^{+} xanthophores percentage that underwent proliferation, cell death, or did not proliferate respectively. n = 11 injected embryos. Significances were calculated using one-way ANOVA followed by Dunnett's multiple comparisons test. Error bars represent means ± SEM.****p<0.0001

(E) Representative images of xanthophore pattern in 28 hpf *csf1a*$^{-/-}$*csf1b*$^{-/-}$, siblings and *csf1ra* mutants. Xanthophore pattern is indicated by WISH staining of *gch2*.

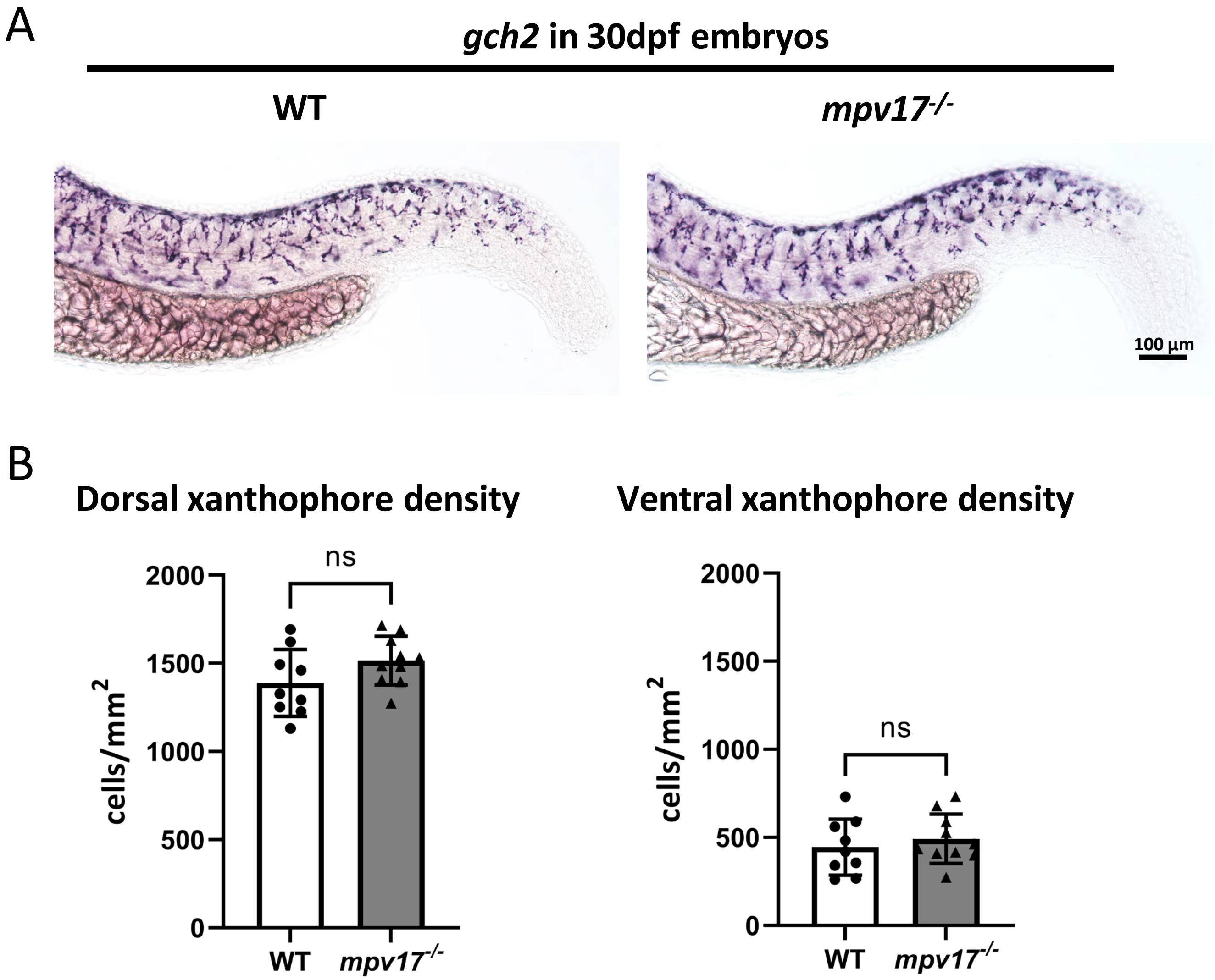

Figure S3. Iridophores are dispensable for xanthophore development and patterning in embryonic zebrafish. Related to Figure 5.

(A and B) Representative images(A) and quantification(B) of xanthophore patterning in the trunk of 30-hpf WT(n=9) and mpv17 mutants(n=10). Error bars represent means ± SD. ns, p>0.05.

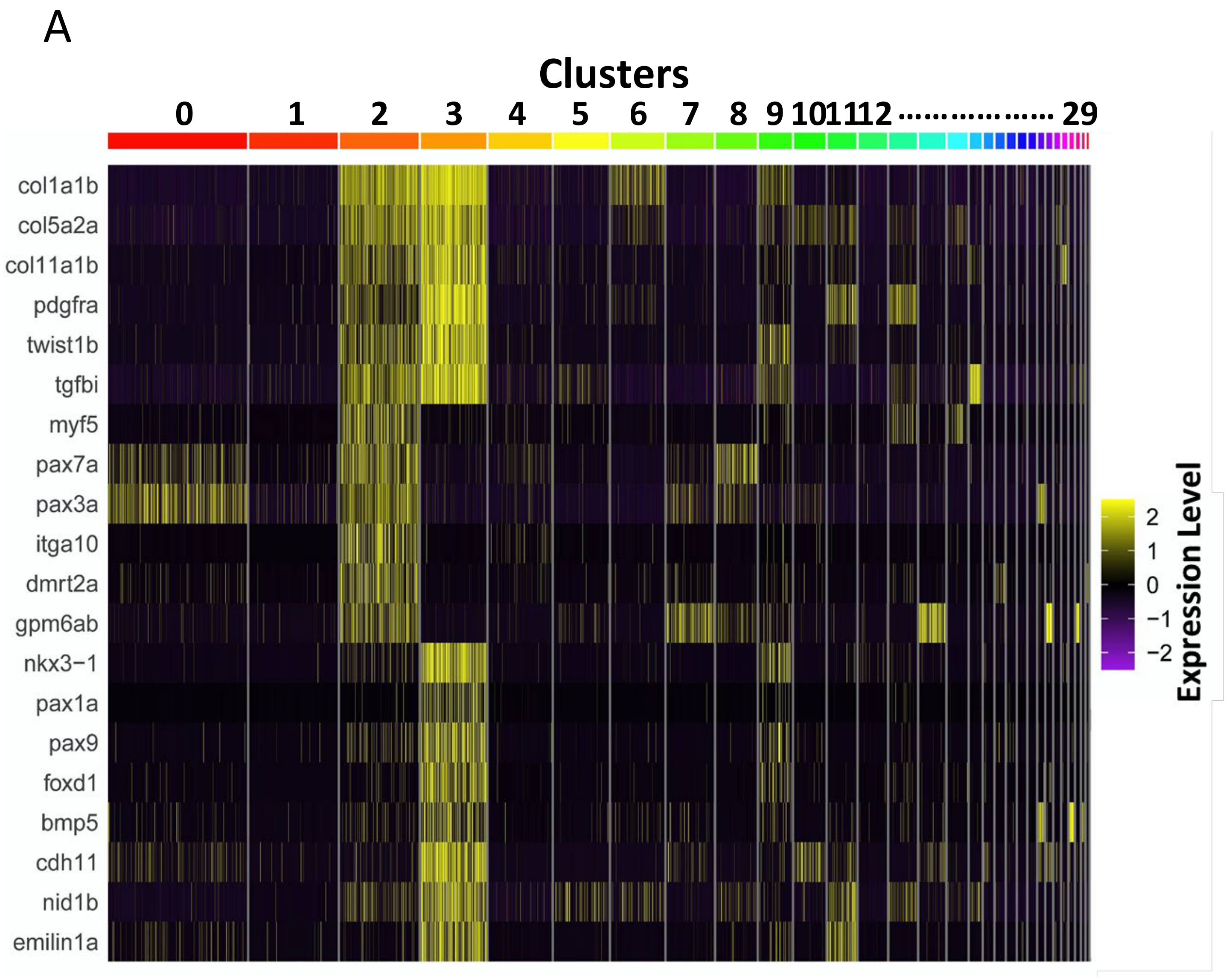

Figure S4—Signature genes of col1a2+ fibroblast and muscle progenitors. Related to Figure 5

(A)Heatmap of 20 differentially expressed genes in Cluster2 and Cluster3

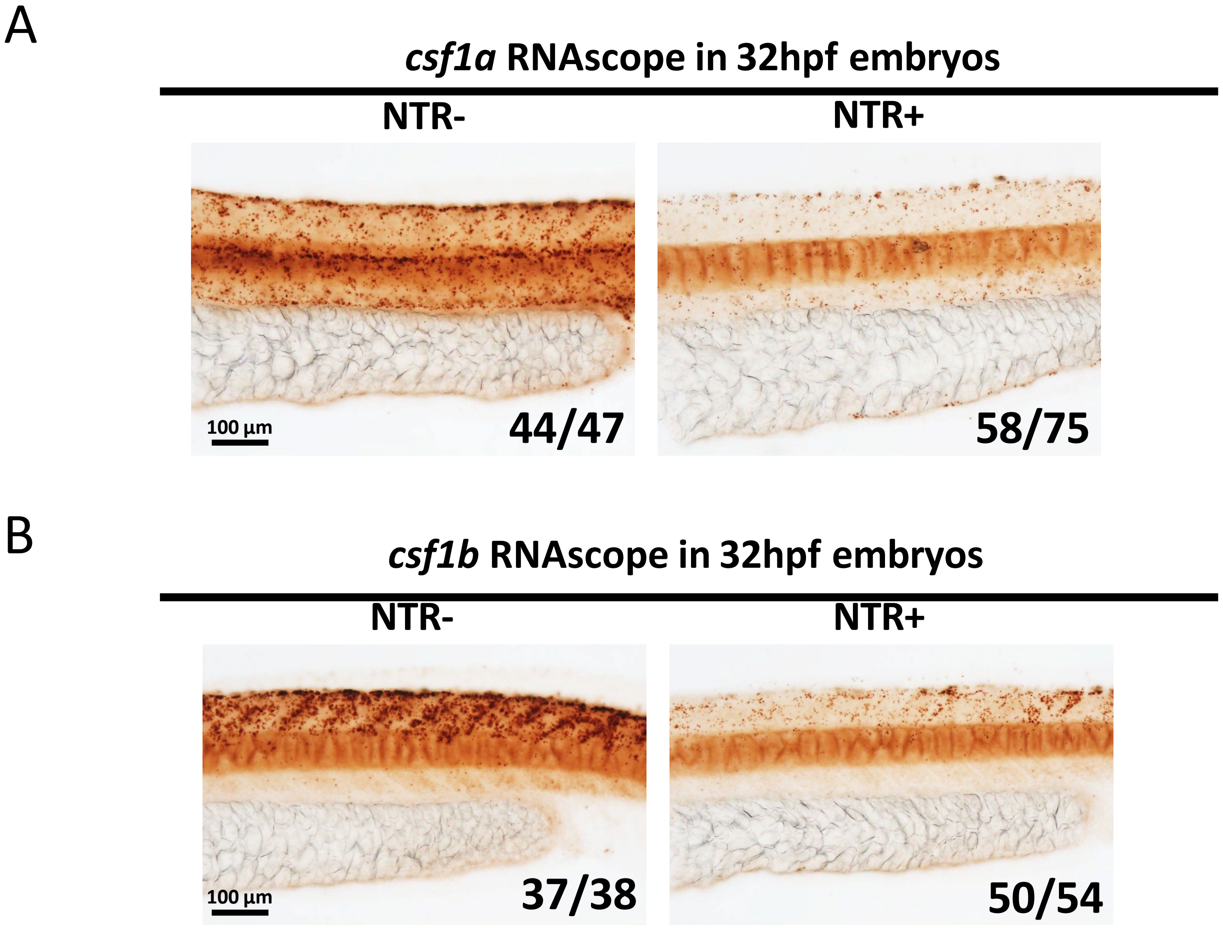

Figure S5. Ablation of *col1a2*$^{+}$ cells reduce *csf1a* and *csf1b* expression in embryonic zebrafish. Related to Figure 6

(A) Representative images of RNAscope detecting *csf1a* RNA granules after ronidazole treatment. Numbers at lower right of each panel indicate the number of embryos with decreased RNAscope signals out of total number of embryos.

(B) Representative images of RNAscope detecting *csf1b* RNA granules after ronidazole treatment. Numbers at lower right of each panel indicate the number of embryos with decreased RNAscope signals out of total number of embryos.

Table S1. List of primers used for genotyping mutants.

**Gene**	**Primer sequences**
csf1a^{hkz9}	Forward primer: CTGCGCCTAATACCCTTTGC<br>Reverse primer: GGACTTTGTTACCTCAATCTCCTC
csf1b^{hkz10}	Forward primer: AAGCATGGCTATGGAAAGCC<br>Reverse primer: CTTGTGCTTGAAGATGGAGC<br>Product digest with TseI
csf1ra^{j4e1}	Forward primer: GGCAGAGCTGTCCTAAAA<br>Reverse primer: CTAGCAAGAGATAAAAGGTGT<br>Product digest with BssSαI
mpv17(roy)	Forward primer: AGGCTCTGATGGCCAAACACCCAT<br>Reverse primer: GAGGTAAGAGTTCCAGACAACAGC

Table S2. List of primers used for real time PCR.

Gene	Primer sequences
csf1a	Forward primer: GTGGACTGGAACTGTGAGGAG<br>Reverse primer: ATGGCTCGAGGATGAAGATGC
csf1b	Forward primer: ACTGCCTACTGCCTCCTAGA<br>Reverse primer: TGGACGTCAAACTGTTTCCGA
eef1a1l1	Forward primer: TACTTCTCAGGCTGACTGTG<br>Reverse primer: ATCTTCTTGATGTATGCGCT

Movie S1. Time-lapse imaging of transiently *TgBAC(csf1ra: eGFP)*-injected embryos from 24-hpf to 32-hpf

White arrows indicated the migrating eGFP+ xanthophores
